# Neuroinflammation: Advancements in Pathophysiology and Therapies

**DOI:** 10.3390/ijms26125664

**Published:** 2025-06-13

**Authors:** David Martín-Hernández

**Affiliations:** 1 Department of Pharmacology and Toxicology, Faculty of Medicine, Complutense University of Madrid (UCM), Hospital 12 de Octubre Research Institute (Imas12), Neurochemistry Research Institute UCM (IUIN), Spanish Network in Stress Research (REIS), 28040 Madrid, Spain; davmar04@ucm.es; 2 Biomedical Network Research Center of Mental Health, Institute of Health Carlos III (CIBERSAM, ISCIII), 28029 Madrid, Spain

Neuroinflammation is a common feature across a wide range of disorders affecting the central nervous system (CNS). While it plays a protective and homeostatic role under physiological conditions, chronic or dysregulated neuroinflammation can become harmful, disrupting CNS function and contributing to disease progression. Increasing evidence also implicates neuroinflammation in physiological processes such as aging, underscoring its complex and context-dependent nature. A major question in the field is whether neuroinflammation acts as a cause, a consequence, or a merely concurrent phenomenon in these disorders. This is far from a trivial issue, as the answer will influence how neuroinflammation should be therapeutically addressed. Regardless of its origin, the presence of uncontrolled and chronic neuroinflammation in the CNS poses a threat to its normal function, since it can exacerbate disease pathophysiology, worsen symptoms, and limit the effectiveness of current treatments. Although we already have robust evidence supporting the efficacy of therapies aimed at modulating neuroinflammation in certain diseases, a deeper understanding of the molecular mechanisms underlying neuroinflammatory processes is crucial. Such knowledge will enhance our comprehension of its role in CNS pathophysiology and pave the way for the development of novel therapeutic strategies.

This Special Issue features eight articles (four original research papers and four reviews) that delve into neuroinflammation across various CNS disorders. These contributions provide new insights into both the mechanisms and pathophysiological relevance of neuroinflammation, as well as the therapeutic potential of its modulation, including novel molecular targets.

Beginning with original research papers, Kupnicka et al. [1] explore the interplay between morphine dependence and long-term perinatal fluoride exposure, with a focus on neuroinflammatory responses in key brain regions such as the prefrontal cortex, striatum, hippocampus, and cerebellum. The authors investigated the expression of inflammatory markers in rats exposed to fluoride from gestation through early development, followed by the induction of morphine dependence. Their findings demonstrate that both morphine and fluoride independently and synergistically influence neuroinflammation. Morphine withdrawal upregulated cycloxigenase-1 (COX-1) and COX-2 expression and activated microglia and astrocytes, as evidenced by increased Iba1 and GFAP levels, respectively. Notably, prior fluoride exposure modified these effects in a region-dependent manner. The results suggest that fluoride, a widespread environmental factor, may exacerbate the inflammatory processes associated with morphine dependence, potentially affecting the dopaminergic and neuroplastic responses involved in addiction and withdrawal. Further research needs to address the direct or indirect mechanisms underlying the observed effects of morphine and fluoride on the measured neuroinflammatory parameters.

De Masi et al. [2] combine the analysis of peripheral blood mononuclear cells (PBMCs) and extracellular vesicles (EVs) in multiple sclerosis (MS). The authors highlight the emerging role of EVs, nanometer-sized membrane-bound vesicles carrying biologically active molecules, as key mediators of intercellular communication and inflammation in MS. Using electron microscopy, they observed significant ultrastructural alterations in PBMCs from MS patients, including increased numbers of multivesicular bodies, autophagosomes, and morphologic features indicative of dysregulated membrane trafficking. Protein analysis of EV cargo revealed a distinct inflammatory molecular profile in MS-derived EVs, with elevated levels of pro-inflammatory and cytoskeletal regulators such as heterodimeric enzyme alpha-glucosidase II (GANAB), interferon-induced protein 35 (IFI35), nuclear factor kappa B (NF-κB), and cofilin 1, suggesting active vesiculation from stressed or activated immune cells. Notably, some proteins were selectively expressed depending on vesicle size (small vs. large), indicating a functional compartmentalization of EV-mediated signaling. Although limited by sample size, the study provides compelling evidence that non-invasive ultrastructural and molecular analysis of PBMCs and plasma-derived EVs can reflect inflammatory status and may contribute to the identification of novel biomarkers or mechanisms in MS.

Bober et al. [3] investigate the pivotal role of chemokine signaling via chemokine receptor 2 (CCR2) and CCR5 in mediating pain hypersensitivity in a streptozotocin-induced mouse model of diabetes, revealing the sex-dependent mRNA upregulation of chemokine ligands (CCLs). Their findings demonstrate that a single administration of cenicriviroc (CVC), a dual CCR2/CCR5 antagonist, produces robust and superior analgesic effects in both male and female diabetic mice compared to the selective antagonists of either CCR2 or CCR5 alone. The authors also examined the effects of the repeated co-administration of CVC and morphine in female mice over time. While CVC delayed the onset of opioid tolerance, the analgesic efficacy of the combination diminished over time and was associated with increased blood glucose levels and weight loss, findings that warrant further investigation. Significantly, the repeated administration of CVC alone produced even greater analgesic effects than morphine alone or in combination with CVC. Future studies will need to evaluate whether these effects are replicated in male mice. Nonetheless, CVC emerges as a promising therapeutic strategy for diabetic neuropathic pain, particularly given its established clinical safety profile.

In the latest published original research article included in this Special Issue, Huynh et al. [4] deal with apolipoprotein E4 (APOE4), a major genetic risk factor for Alzheimer’s disease (AD), particularly in aging individuals. APOE4 disrupts cholesterol metabolism and leads to the accumulation of cholesteryl esters (CEs) in the brain, contributing to neuroinflammation. The authors propose a therapeutic strategy based on inhibiting the cholesterol storage enzyme acyl-CoA:cholesterol acyltransferase 1 (ACAT1), which is responsible for CE synthesis, using the nanoparticle formulation F12511 in both primary microglial cultures and in vivo in aged APOE4 mice. Their findings demonstrate that the pharmacological inhibition of ACAT1 with F12511 effectively reduces the formation of CE-rich lipid droplets, activates the ATP-binding cassette transporter A1 (ABCA1), and attenuates neuroinflammatory signaling by modulating Toll-like receptor 4 (TLR4)-dependent pathways. The therapeutic effects were more pronounced in aged mice, reflecting the heightened vulnerability associated with aging in human APOE4 carriers. Importantly, the beneficial effects were observed even in the absence of amyloid-beta (Aβ) pathology, extending the potential utility of this intervention to early or preclinical stages of AD. Future studies validating these findings in human induced pluripotent stem cell (iPSC) models and across diverse CNS cell types could pave the way for clinical translation.

Among the review articles, Sanz et al. [5] provide a comprehensive overview of the neuroinflammatory process, offering a detailed description of the various cell types involved (astrocytes, microglia, and infiltrating peripheral immune cells) and presenting evidence of their individual contributions to epilepsy. The authors emphasize the bidirectional relationship between neuroinflammation and epilepsy, wherein each can trigger and exacerbate the other, reinforcing their mutual causative and consequential roles. Beyond conventional anti-inflammatory agents targeting the immune system, the review focuses on compounds originally developed for other medical conditions that exhibit anti-inflammatory properties, supporting their potential for repurposing in epilepsy treatment. These include the antidiabetic drug metformin, the immunomodulators fingolimod and dimethyl fumarate approved for MS, the antihypertensive propranolol, the nonsteroidal anti-inflammatory drug ibuprofen, and the antioxidant N-acetylcysteine. Such agents may offer therapeutic benefit either as monotherapies or as adjuvants to current antiepileptic drugs, providing new avenues for the pharmacological management of epilepsy.

Romero-Ramírez et al. [6] explore the emerging role of bile acids (BAs) in neuroinflammation, not only due to their ability to cross the blood–brain barrier (BBB), but also due to the possible existence (hypothetical and not yet confirmed) of endogenous BA metabolism within the CNS. The enzymes involved in cholesterol metabolism, such as CYP27A1 and CYP46A1, are expressed in the brain, which also harbors receptors, enzymes, and molecules capable of mediating or modulating BA signaling, most notably the Takeda G protein-coupled receptor 5 (TGR5). The authors discuss the involvement of BAs in neurodegenerative and psychiatric disorders, the efficacy of BA-based interventions in these contexts, and the molecular pathways that may mediate their effects (anti-inflammatory cAMP production, endoplasmic reticulum stress modulation, reduced apoptosis and mitochondrial dysfunction, chaperone activity, and direct effects on neural cells). They emphasize the importance of comparing the preclinical results of BA administration with those of established anti-inflammatory agents before advancing to clinical trials, including studies involving dietary interventions.

Rather than focusing on a specific disease, the review by Jurcau et al. [7] addresses the close relationship between neuroinflammation, systemic inflammation, and aging. Following a thorough examination of aging-related footprints in CNS cell populations, gray and white matter volumes, BBB, and the immune system, the authors detail alterations in the key inflammatory signaling pathways associated with neuroinflammation in the aging brain, including NF-κB, tumor necrosis factor-alpha (TNF-α), reactive oxygen species (ROS), receptor for advanced glycation end-products (RAGE), cyclic GMP-AMP synthase/stimulator of interferon genes (cGAS/STING), inflammasome, and necroptosis. Potential triggers such as circadian rhythm disruption, alterations in gut microbiota, changes in cholinergic neurotransmission, and glial cell dysfunction (with an emphasis on sex differences) are also discussed. This exhaustive review compiles the consequences of “inflammaging” (a term denoting the intersection of inflammation and aging), current approaches for its detection through imaging and biomarkers, and potential therapeutic strategies aimed at its mitigation. The authors also critically assess the limitations of the existing research and propose future directions, including the development of non-invasive biomarkers, more specific therapeutic targets, personalized medicine approaches, and enhanced collaboration between basic science, clinical research, and industry.

Finally, Popescu et al. [8] compile the current evidence on the involvement of the gut and oral microbiome in AD. After outlining the neuroinflammatory mechanisms associated with AD, the authors provide an in-depth discussion of how the microbiome may influence disease pathophysiology through immune system modulation, the production of CNS-active compounds, barrier dysfunction, and shifts in microbial composition. They highlight the anti-inflammatory and neuroprotective potential of nutraceuticals such as quercetin, curcumin, BAs (reviewed in detail in another article from this collection), and polyunsaturated fatty acids (PUFAs). A particularly compelling section is devoted to sex-related differences in the microbiome, with a focus on menopause and hormones such as testosterone. Both preclinical and clinical evidence supports the possible role of these factors in AD pathogenesis. However, the authors acknowledge the limitations stemming from inter-study, population, and methodological variability. Longitudinal studies, microbiota-targeted interventions, and integration with classical AD biomarkers will be essential to deepen our understanding of the therapeutic potential of microbiome modulation in this neurodegenerative disorder.

Altogether, this Special Issue underscores the significance of expanding our knowledge on the pathophysiological mechanisms of neuroinflammation across diverse CNS diseases. The studies compiled here illustrate the efficacy of both established and novel anti-inflammatory therapies in modulating neuroinflammatory processes, contributing to alleviating symptomatology, and opening up new therapeutic avenues.
